# Psychometric evaluation of a patient-reported outcomes instrument for congenital thrombotic thrombocytopenic purpura

**DOI:** 10.1186/s41687-023-00592-w

**Published:** 2023-07-14

**Authors:** Abiola Oladapo, Diane Ito, Ana María Rodriguez, Stephanie Philpott, Robert Krupnick, Veleka Allen, Christopher Hibbard, Marie Scully, Bruce Ewenstein

**Affiliations:** 1 Takeda Development Center Americas, Inc., Cambridge, MA USA; 2 IQVIA, Madrid, Spain; 3 IQVIA, Reading, UK; 4 IQVIA, Cambridge, MA USA; 5 IQVIA, New York, NY USA; 6 Department of Haematology, University College London Hospitals, London, UK

**Keywords:** Congenital thrombotic thrombocytopenic purpura, TTP, Rare diseases, Psychometrics, Patient-reported outcome measures, PRO, Clinical outcome assessment, Quality of life

## Abstract

**Background:**

Congenital thrombotic thrombocytopenic purpura (cTTP) is an ultra-rare, life-threatening hereditary disorder that causes patients to experience significant morbidity and decreased health-related quality of life (HRQoL). A cTTP disease-specific patient-reported outcome (PRO) instrument that is reflective of patients’ experiences with the disorder does not currently exist. The objective of this study was to evaluate and validate the psychometric properties of the Congenital Thrombotic Thrombocytopenic Purpura–Patient Experience Questionnaire (cTTP-PEQ), developed using a literature review, interviews with expert clinicians, and qualitative concept elicitation and cognitive debriefing interviews.

**Methods:**

This prospective, observational study (NCT03519672) was conducted with patients diagnosed with cTTP currently receiving treatment. Patients were enrolled through investigator sites and direct-to-patient recruitment. Individuals completed electronic self-administered PRO measures, including the cTTP-PEQ, at baseline and Day 14 (+ up to 10 days). The cTTP-PEQ consisted of five multi-item domains (Pain/Bruising, Cognitive Impairment, Visual Impairment, Mood, Treatment Burden) and three single-item domains (Fatigue, Headache, Activity Limitation), and assessed symptoms and impact of cTTP in the previous 24 h, 7 days, and 2 weeks. Convergent and discriminant validity were evaluated using Spearman’s rank correlation coefficients. Known-groups validity was assessed between patient groups separated by Patient Global Impression of Severity (PGI-S; normal vs. mild/moderate/severe). Internal reliability was assessed using Cronbach’s alpha. Test–retest reliability was assessed using intraclass correlation coefficients (ICCs).

**Results:**

Thirty-six patients participated in this study. Convergent validity was confirmed with high-to-moderate correlations (*r* ≥ 0.4) for 12/15 hypothesized relationships between pairs of domains and/or total scores. Discriminant validity was confirmed with low correlations (*r* < 0.3) observed for 5/7 hypothesized relationships. Known-groups validity was confirmed with significant differences (*p* ≤ 0.05) in mean cTTP-PEQ scores between the two PGI-S groups for most domains and items at both timepoints. Cronbach’s alpha was 0.88 at baseline and 0.91 at Day 14, confirming internal consistency of the instrument. Test–retest reliability was also confirmed with a high ICC (0.96).

**Conclusion:**

This study validates the psychometric properties of the novel cTTP-PEQ for use in research and clinical practice to assess HRQoL among patients with cTTP. This instrument will be particularly useful when assessing cTTP disease burden and the impact of new treatments.

**Supplementary Information:**

The online version contains supplementary material available at 10.1186/s41687-023-00592-w.

## Introduction

Thrombotic thrombocytopenic purpura (TTP) is a rare, life-threatening blood disorder resulting from a deficiency in the clotting enzyme ADAMTS13 (a disintegrin and metalloproteinase with a thrombospondin type 1 motif, member 13) [1, 2]. This leads to clot formation in small blood vessels, including those in the brain, heart, and kidneys [1, 2]. The hereditary form of the disorder, known as congenital (c)TTP, is caused by a mutation in the gene encoding ADAMTS13 [3–5] and is considered an ultra-rare disease with an estimated prevalence of one case per million [3, 4, 6–8].

The spectrum of severity of cTTP can be extremely varied, including asymptomatic disease, single episode and chronic-relapsing forms, and multiorgan failure [6]. The classic presentation of cTTP includes thrombocytopenia, microangiopathic hemolytic anemia, and varying degrees of end organ damage [1, 6]. There appear to be two disease presentation peaks, with approximately 40% of patients experiencing symptom onset during the neonatal/childhood period and approximately 60% in adulthood, typically related to pregnancy [9]. If left untreated, acute cTTP episodes are associated with high morbidity and mortality [10]. Even in the absence of acute attacks, patients with cTTP are at a higher risk of thrombotic events, such as myocardial infarction or stroke, and persistent renal or neurologic abnormalities [3, 9, 11]. Patients experience numerous disease-related complications, and patient-reported complaints often include fatigue, headache, abdominal pain, bruising, cognitive impairment, and experience of depression and mood alterations [9, 12–15].

Although there is no specific therapy approved for TTP treatment, many patients require ongoing prophylactic treatment with intravenous plasma infusion, typically administered every 2 to 3 weeks to help alleviate symptoms and reduce long-term complications [9, 13]. von Willebrand factor (VWF) and factor VIII (FVIII) concentrates have also been used to treat TTP, but levels of ADAMTS13 can remain low and inconsistent [16]. Furthermore, plasma infusion can be associated with volume overload and significant complications, such as an allergic reaction to, or the risk of viral transmission from, the donor blood [17, 18]. In addition, most patients will require lifelong treatment, necessitating frequent travel to available facilities to receive infusions. Treatment-related adverse events and associated limitations can lead to substantial discomfort and distress for patients and contribute to the overall burden of the disease, negatively impacting patients’ health-related quality of life (HRQoL) [12].

Accurate assessment of disease- and treatment-related burden is essential to improve our understanding of the effect of cTTP on patients’ HRQoL and to assess the impact of new treatments. Therefore, it is important that any patient-reported outcome (PRO) instrument measures the concepts most relevant to a patient’s specific disease condition and treatment. Several generic PRO instruments have been used to assess the HRQoL of patients with TTP. These include using the Medical Outcomes Study 36-Item Short-Form Health Survey (SF-36) to assess physical and cognitive long-term deficits in HRQoL after recovery [19], and the Headache Impact Test (HIT-6) survey to assess the impact of the frequency and severity of headaches in patients with TTP [20]. In addition, HRQoL measures exist that are validated for use in other blood disorders, such as the Haemophilia Quality of Life Questionnaire for Adults (A36 Hemofilia-QoL) and the Haemophilia Quality of Life Questionnaire for Children (Haemo-QoL) [21, 22]. However, these instruments are not specific to cTTP and do not capture the full spectrum of the cTTP disease burden. There have been limited studies investigating HRQoL in patients who specifically have cTTP, and there are currently no existing instruments developed for use in the cTTP patient population.

Therefore, a PRO instrument that was comprehensive and specific for cTTP, whilst having a minimal burden on the patient to complete, would be valuable for the clinical management of cTTP. A conceptual model capturing the symptoms and impacts of cTTP has previously been formulated [12]. This model served as the foundation for generating a draft instrument, the Congenital Thrombotic Thrombocytopenic Purpura–Patient Experience Questionnaire (cTTP-PEQ), that is reflective of the most salient aspects of the experience of patients with this disorder. This instrument was developed and tested with patients living with cTTP and further refined for the purpose of psychometric validation. The objective of the present study was to assess the psychometric properties of the cTTP-PEQ and validate the tool against existing PRO measures in patients with cTTP.

## Methods

### The Congenital Thrombotic Thrombocytopenic Purpura–Patient Experience Questionnaire

Concept elicitation and development of a cTTP-specific PRO instrument was conducted in accordance with good practice guidance for establishing content validity [23]. The conceptual model was established following a comprehensive literature search, interviews with expert clinicians, and patient concept elicitation interviews [12]. This resulted in an initial pool of 35 items. Preliminary assessment of content validity was conducted with ten adult patients across two iterated rounds of 60-minute cognitive debriefing telephone interviews. The final cTTP-PEQ was revised to include 26 items covering disease symptoms, the impact of cTTP, and treatment-related items. The final items were designed to assess the patient’s experience of fatigue and joint, muscle, abdominal, and chest pain in the previous 24 h; headache, cognitive and visual impairments, bruising, feelings of depression and mood alterations, and activity limitation in the past 7 days; and patients’ attitudes, experienced side effects, work/school absences, and travel impact associated with cTTP treatment received during the previous 2 weeks (Table S1). The first 20 items were answered by all patients, and Items 21 to 26 were answered only by patients who received cTTP treatment within the last 2 weeks.

**Fig. 1:**
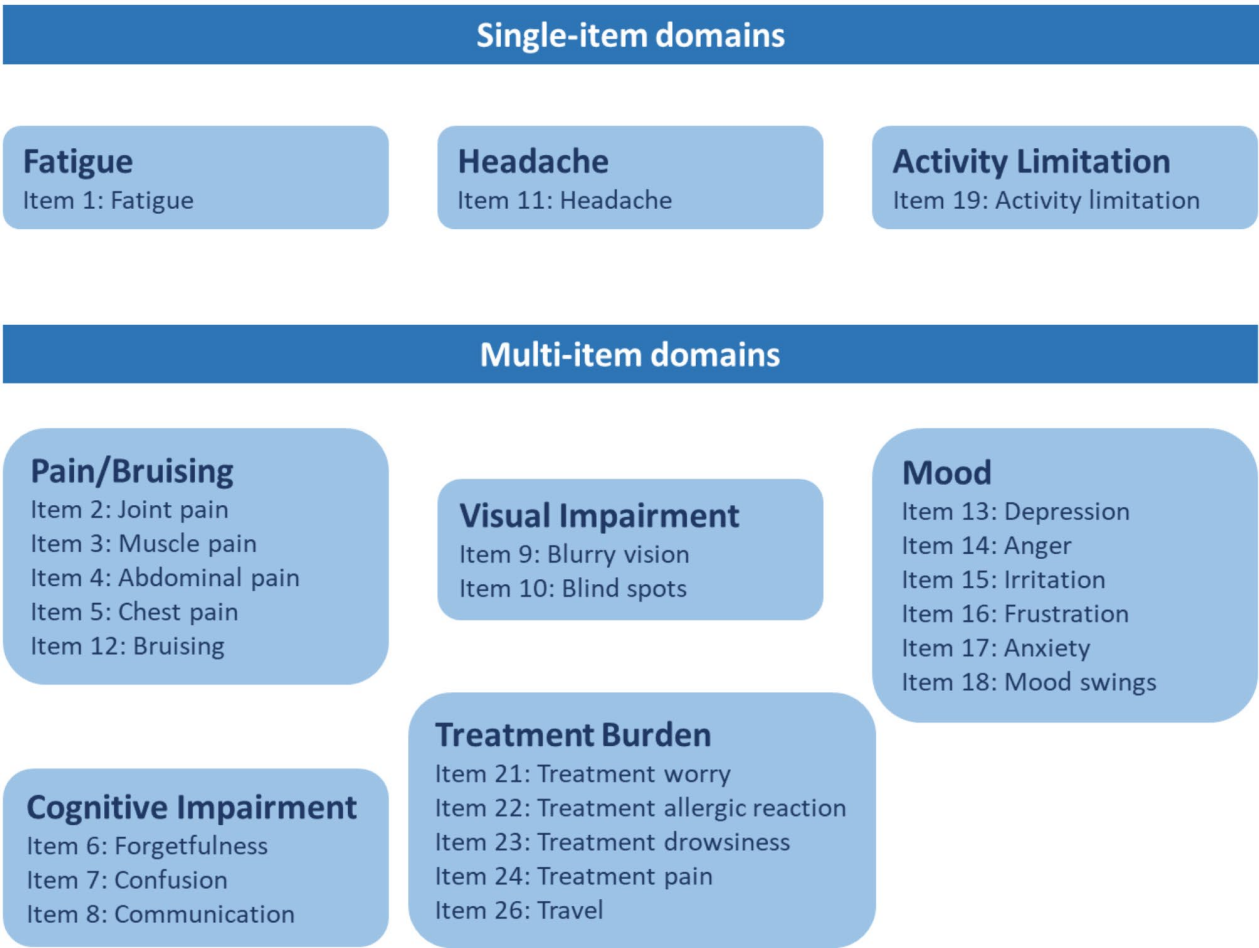
Composition of the cTTP-PEQ multi-item and single-item domains Domains are based on a conceptual model because the small sample size did not allow for domains to be constructed using factor analysis *cTTP-PEQ* Congenital Thrombotic Thrombocytopenic Purpura–Patient Experience Questionnaire

A scoring algorithm for the cTTP-PEQ was developed, with most items scored using a numerical scale ranging from 0 to 4, 0 to 5, or 0 to 10 (Table S1). Fatigue, Headache, and Activity Limitation were scored as single items. The remaining items were summed into five multi-item domains, which were conceptually developed based on whether the items address similar concepts as informed by the literature review and patient interviews used. Due to the small sample size of the study, it was not possible to carry out a factor analysis to construct the cTTP-PEQ domains. Therefore, the domains described in this study are based on the multi-item concepts from the previously described conceptual model (Fig. 1). The cTTP-PEQ total score was calculated on the basis of the sum of all items (0–152), with a higher score indicating greater burden. Items 20 and 25 were excluded from the domain and total scoring, and from most of the psychometric testing and correlation analyses. Item 20 was a conditional item for the inclusion of additional items (Items 21 to 26) and Item 25 did not measure a concept represented in the conceptual model.

### Study design

The cTTP-PEQ was evaluated in a prospective, non-interventional, observational cohort study (NCT03519672) conducted at three study sites that treat patients with rare hematologic conditions between July 2018 and April 2019. Two of the study sites were in the United Kingdom (University College London Hospitals, London, UK and Cambridge University NHS Foundation Trust–Addenbrooke’s Hospital, Cambridge, UK) and one was in the United States (The Methodist Hospital, Houston, TX, USA). Owing to the inherent difficulties associated with recruiting a representative sample of patients with cTTP, patients were also enrolled by direct-to-patient recruitment using two third-party qualified vendors that specialize in recruiting patients with rare diseases, facilitated by a contract research organization. Adult (≥ 18 years of age) and adolescent (12–17 years of age) patients with the ability to read, write, and speak English were eligible for inclusion. Eligible patients had a diagnosis of cTTP and were currently receiving prophylactic or on-demand treatment with fresh frozen plasma, solvent detergent plasma, or VWF/FVIII concentrate. Patients participating in a concurrent clinical trial were excluded from the study. All patients provided informed consent for participation, and were screened and followed up using the same process, regardless of recruitment method. For patients recruited using the direct-to-patient approach, verification of the cTTP diagnosis was provided by the patient’s treating physician.

Following recruitment, information on patients’ sociodemographic characteristics and cTTP-related clinical measures, including disease severity, presence and number of comorbidities, and cTTP treatment history, were recorded. Using either the study site or their own internet-connected computer or device, each patient completed eight self-administered, electronically formatted PRO measures, including the cTTP-PEQ. As patients receiving prophylactic plasma infusions typically undergo treatment every 2 to 3 weeks, a repeat testing interval of 14 days (+ up to 10 days) from baseline was selected. Patients completed the same set of electronically formatted PRO measures at Day 14 (+ 10 days) following their first assessment. Changes in patient health status from baseline were also assessed using the Patient Global Impression of Change (PGI-C) scale [24].

### Additional outcome measures and procedures

In addition to the cTTP-PEQ, patients completed eight existing PRO measures to assess the psychometric properties of the newly developed cTTP-specific instrument. These included the Patient-Reported Outcomes Measurement Information System (PROMIS®)-29 Profile [25], the HIT-6 [26], the Condensed Molecular and Clinical Markers for the Diagnosis and Management (MCMDM) of von Willebrand Disease Bleeding Questionnaire: Bruising Subscale [27], the Perceived Deficits Questionnaire–5 items (PDQ-5) [28], the 25-item National Eye Institute Visual Function Questionnaire (NEI-VFQ-25) [29], and the Work Productivity and Activity Impairment Questionnaire (WPAI-GH). These measures were selected based on their conceptual resemblance or dissonance to the cTTP-PEQ domains and were used to assess convergent and discriminant validity [30]. For adolescent patients the PROMIS® pediatric measures and WPAI-GH plus classroom impairment questions were used as alternatives to the adult versions. In addition, the PGI-C scale and the Patient Global Impression of Severity (PGI-S) scale [31] were used to assess known-groups validity and test–retest reliability.

### Statistical analysis

Quantitative data from patient characteristics and each individual PRO measure were summarized using descriptive statistics, which were calculated for each item, subscale or domain score, and total score, when applicable, at baseline and Day 14. Due to the non-interventional nature of the study, missing values were expected for the second assessment owing to patient drop-out. Patients who did not complete the second assessment, despite multiple reminders, were assumed missing at random. Missing and invalid observations were recorded as a separate category, and missing data were not imputed. Percentages of patients were based on the number of patients who completed all items. Analyses were based on the available data for item-specific analyses and on observations with full data for multi-item domains and totals or multiple timepoints.

#### Validity

To assess the strength of hypothesized relationships between the cTTP-PEQ and the existing PRO measures, Spearman’s rank correlation coefficients were used to evaluate convergent and discriminant validity at baseline and Day 14. The hypothesized relationships between the cTTP-PEQ domains and existing PRO measures expected to demonstrate “moderate-to-high” or “low” correlation are detailed in Table S2. Correlation coefficient values ≤ 0.19 were considered “very low,” values between 0.20 and 0.29 were considered “low,” values between 0.30 and 0.49 were considered “moderate,” and those between 0.50 and 0.69 were considered “high.” Correlation coefficients ≥ 0.70 were considered “very high” [32].

Known-groups validity was assessed at baseline and on Day 14 by comparing the mean cTTP-PEQ scores between patient groups separated by PGI-S score (normal vs. mild/moderate/severe). Significance was defined as an alpha value ≤ 0.05.

#### Reliability

Item-to-item and item-to-total correlations, estimated using Spearman’s rank correlation coefficients, were used to support the grouping of items measuring a similar construct in order to assess the internal reliability of the conceptual model. Internal consistency was assessed using Cronbach’s alpha, which was calculated for the total cTTP-PEQ score and for all subscales separately. Values of 0.70–0.95 were considered adequate for group-level comparisons. Test–retest reliability was assessed for each domain and total score of the cTTP-PEQ by calculating the intraclass correlation coefficient (ICC) between baseline and Day 14 for patients who reported no change in PGI-C score. Values of 0.4–0.75 were considered to represent fair-to-good reliability and values > 0.75 represent excellent reliability.

## Results

### Patients

A total of 38 patients were enrolled in the study. Two of these patients were adolescents and 36 were adults. Owing to the small sample size of the adolescent population, these patients were excluded from the analysis, leaving a total of 36 adult patients in the full analysis population. Baseline characteristics are shown in Table 1. The majority of patients were female (66.7%) with a mean (standard deviation) age of 36.3 (13.2) years.

**Table 1:** Baseline Demographic and Clinical Characteristics

	Population Sample (*N* = 36)	
	Missing, *n*	Patients
**Sex, n (%)**	0	
Male		12.0 (33.3)
Female		24.0 (66.7)
**Age, years**	1	
Mean (SD)		36.3 (13.2)
Median (range)		35.5 (0^a^–70)
**Race, n (%)**	0	
American Indian or Alaska Native		0
Asian		1 (2.8)
Black or African American		1 (2.8)
Native Hawaiian or other Pacific Islander		0
White		34 (94.4)
**Employment/educational status, n (%)**	0	
Employed (full time or part time)		23 (3.9)
School (full time or part time)		2 (5.6)
Unemployed		4 (11.1)
Retired		2 (5.6)
Disabled		3 (8.3)
Other		2 (5.6)
**Clinician-reported cTTP severity, n (%)**	13	
Normal		0
Mild		13 (56.5)
Moderate		8 (34.8)
Severe		2 (8.7)
**Any comorbidities, n (%)**	13	
No		18 (78.3)
Yes		5 (21.7)
**Number of comorbidities, n (%)**	13	
0		18 (78.3)
1		3 (13.0)
2		1 (4.3)
3		0
4		1 (4.3)
**cTTP treatment history (treatment was received), n (%)**	23	
Periodically		9 (69.2)
As needed		4 (30.8)
**cTTP treatment type, n (%)**	0	
Fresh frozen plasma		16 (44.4)
Solvent detergent plasma		13 (36.1)
VWF/FVIII concentrate		6 (16.7)
Unknown		1 (2.8)
**cTTP treatment, n (%)**	0	
Prophylactic		28 (77.8)
On-demand		8 (22.2)

^a^One participant incorrectly put current year as birth year resulting in a minimum age of 0 years

*cTTP* Congenital thrombotic thrombocytopenic purpura, *FVIII* Factor VIII, *SD* Standard deviation, *VWF* von Willebrand factor

### PRO instrument distribution characteristics

Descriptive statistics for the cTTP-PEQ item and domain scores at baseline and Day 14 are detailed in Table 2. Of the 36 patients included in the study, 30 (83.3%) patients completed cTTP-PEQ items 1–20 at baseline and 29 (80.6%) patients completed items 1–20 at Day 14. Missingness patterns were explored and no observable patterns were noted. As a result, data were assumed to be missing at random. For Item 20, which assessed whether patients had received treatment for cTTP in the past 2 weeks, 27 of 36 (75.0%) patients responded “yes” at baseline, whereas 23 of 36 (63.9%) patients responded “yes” on Day 14. Of those patients who had received treatment in the last 2 weeks, 18 (66.7%) provided answers to Items 21–26 at baseline and all 23 (100.0%) patients provided answers to these items on Day 14. The descriptive statistics for the PROMIS-29 Profile, HIT-6, MCMDM Bleeding Questionnaire Bruising Subscale, PDQ-5, NEI-VFQ-25, and WPAI-GH PRO instruments, measured at baseline and Day 14, are detailed in Table S3.

**Table 2:** Descriptive Statistics for the cTTP-PEQ Items, Domain Score, and Total Score

cTTP-PEQ Item (Score Range) or Domain	*N* = 36						Change from Baseline (SD)^b^
	Baseline			Day 14			
	Missing, *n*	Mean (SD) or *n* (%)^a^	Range	Missing, *n*	Mean (SD) or *n* (%)^a^	Range	
**Item 1. Fatigue (0–10)**	1	4.37 (3.04)	0–10	5	3.55 (2.71)	0–9	– 0.87 (2.49)
**Item 2. Joint pain (0–10)**	1	2.14 (2.42)	0–8	4	2.19 (2.33)	0–7	0.09 (1.67)
**Item 3. Muscle pain (0–10)**	1	1.71 (2.49)	0–10	4	1.78 (2.39)	0–8	– 0.03 (1.53)
**Item 4. Abdominal pain (0–10)**	1	1.63 (2.84)	0–9	4	1.63 (2.71)	0–10	– 0.09 (1.65)
**Item 5. Chest pain (0–10)**	1	0.60 (1.54)	0–6	4	0.53 (1.32)	0–5	– 0.13 (1.79)
**Item 6. Forgetfulness**	1			4			0.00 (0.72)
None of the time		8 (22.9)			9 (28.1)		
A little of the time		10 (28.6)			9 (28.1)		
Some of the time		9 (25.7)			5 (15.6)		
A good bit of the time		8 (22.9)			7 (21.9)		
Most of the time		0			2 (6.3)		
All of the time		0			0		
**Item 7. Confusion**	1			4			– 0.13 (0.49)
None of the time		20 (57.1)			19 (59.4)		
A little of the time		6 (17.1)			7 (21.9)		
Some of the time		5 (14.3)			3 (9.4)		
A good bit of the time		4 (11.4)			3 (9.4)		
Most of the time		0			0		
All of the time		0			0		
**Item 8. Difficulty communicating**	1			4			– 0.25 (0.76)
None of the time		11 (31.4)			12 (37.5)		
A little of the time		11 (31.4)			11 (34.4)		
Some of the time		6 (17.1)			5 (15.6)		
A good bit of the time		6 (17.1)			3 (9.4)		
Most of the time		0			1 (3.1)		
All of the time		1 (2.9)			0		
**Item 9. Blurry vision**	1			4			– 0.03 (0.93)
None of the time		18 (51.4)			18 (56.3)		
A little of the time		12 (34.3)			7 (21.9)		
Some of the time		1 (2.9)			3 (9.4)		
A good bit of the time		1 (2.9)			2 (6.3)		
Most of the time		2 (5.7)			1 (3.1)		
All of the time		1 (2.9)			1 (3.1)		
**Item 10. Experiencing blind spots**	1			4			0.13 (0.55)
None of the time		23 (65.7)			23 (71.9)		
A little of the time		7 (20.0)			6 (18.8)		
Some of the time		2 (5.7)			0		
A good bit of the time		1 (2.9)			1 (3.1)		
Most of the time		0			0		
All of the time		2 (5.7)			2 (6.3)		
**Item 11. Headache**	1			4			– 0.03 (0.70)
None of the time		12 (34.3)			12 (37.5)		
A little of the time		12 (34.3)			9 (28.1)		
Some of the time		6 (17.1)			6 (18.8)		
A good bit of the time		1 (2.9)			3 (9.4)		
Most of the time		3 (8.6)			1 (3.1)		
All of the time		1 (2.9)			1 (3.1)		
**Item 12. Bruising (0–10)**	1	1.49 (2.22)	0–9	4	1.63 (2.41)	0–8	0.09 (1.28)
**Item 13. Feeling depressed**	2			4			– 0.13 (0.89)
None of the time		16 (47.1)			12 (37.5)		
A little of the time		8 (23.5)			14 (43.8)		
Some of the time		6 (17.6)			2 (6.3)		
A good bit of the time		2 (5.9)			2 (6.3)		
Most of the time		2 (5.9)			1 (3.1)		
All of the time		0			1 (3.1)		
**Item 14. Anger**	1			5			– 0.03 (1.05)
None of the time		12 (34.3)			13 (41.9)		
A little of the time		13 (37.1)			11 (35.5)		
Some of the time		9 (25.7)			3 (9.7)		
A good bit of the time		1 (2.9)			3 (9.7)		
Most of the time		0			1 (3.2)		
All of the time		0			0		
**Item 15. Irritable**	1			5			0.03 (0.91)
None of the time		5 (14.3)			5 (16.1)		
A little of the time		15 (42.9)			15 (48.4)		
Some of the time		10 (28.6)			5 (16.1)		
A good bit of the time		5 (14.3)			5 (16.1)		
Most of the time		0			1 (3.2)		
All of the time		0			0		
**Item 16. Frustrated**	2			4			0.16 (1.05)
None of the time		9 (26.5)			8 (25.0)		
A little of the time		12 (35.3)			11 (34.4)		
Some of the time		8 (23.5)			5 (15.6)		
A good bit of the time		4 (11.8)			7 (21.9)		
Most of the time		0			0		
All of the time		1 (2.9)			1 (3.1)		
**Item 17. Anxiety**	1			4			– 0.50 (0.916)
None of the time		12 (34.3)			13 (40.6)		
A little of the time		5 (14.3)			8 (25.0)		
Some of the time		7 (20.0)			6 (18.8)		
A good bit of the time		7 (20.0)			4 (12.5)		
Most of the time		4 (11.4)			1 (3.1)		
All of the time		0			0		
**Item 18. Mood swings**	1			4			– 0.13 (1.01)
None of the time		13 (37.1)			16 (50.0)		
A little of the time		12 (34.3)			8 (25.0)		
Some of the time		5 (14.3)			3 (9.4)		
A good bit of the time		4 (11.4)			4 (12.5)		
Most of the time		1 (2.9)			0		
All of the time		0			1 (3.1)		
**Item 19. Activity limitation**	3			13			– 0.13 (0.78)
Not at all		16 (48.5)			14 (60.9)		
A little bit		11 (33.3)			4 (17.4)		
Moderately		2 (6.1)			2 (8.7)		
Quite a bit		4 (12.1)			3 (13.0)		
Extremely		0			0		
**Item 20. TTP treatment**	2			5			NC
No		7 (20.6)			8 (25.8)		
Yes		27 (79.4)			23 (74.2)		
**Item 21. Treatment worry**	10			13			0.09 (0.53)
Not at all		18 (69.2)			14 (60.9)		
A little bit		3 (11.5)			4 (17.4)		
Moderately		1 (3.8)			2 (8.7)		
Quite a bit		3 (11.5)			3 (13.0)		
Extremely		1 (3.8)			0		
**Item 22. Allergic reaction to treatment (0–10)**	9	0.78 (1.60)	0–5	13	0.43 (1.20)	0–5	– 0.13 (1.58)
**Item 23. Drowsiness due to treatment (0–10)**	9	3.26 (3.30)	0–8	13	2.74 (2.97)	0–8	– 0.26 (1.63)
**Item 24. Treatment pain (0–10)**	9	1.85 (2.46)	0–9	13	2.00 (2.71)	0–20	0.17 (1.37)
**Item 25a. Work/school missed for treatment-related reason?**	1			4			NC
No		23 (65.7)			20 (62.5)		
Yes		12 (34.3)			12 (37.5)		
**Item 25b. Work outside of the home/attend school?**	1			4			NC
No		24 (68.6)			25 (78.1)		
Yes		11 (31.4)			7 (21.9)		
**Item 25c. Hours of work/school missed in past 2 weeks**	9	1.78 (3.25)	0–12	13	1.61 (2.71)	0–8	– 0.30 (2.55)
**Item 26. Travel burden**	9			13			0.00 (0.85)
Not at all		12 (44.4)			9 (39.1)		
A little bit		6 (22.2)			6 (26.1)		
Moderately		4 (14.8)			6 (26.1)		
Quite a bit		3 (11.1)			1 (4.3)		
Extremely		2 (7.4)			1 (4.3)		
**Domain score**							
Fatigue (Item 1)	1	4.37 (3.04)	0–10	5	3.55 (2.71)	0–9	– 0.87 (2.49)
Headache (Item 11)	1	1.26 (1.36)	0–5	4	1.22 (1.31)	0–5	– 0.03 (0.70)
Activity Limitation (Item 19)	3	0.82 (1.01)	0–3	4	0.75 (1.16)	0–4	– 0.13 (0.78)
Pain/Bruising (Items 2, 3, 4, 5, and 12; score range 0–45)	1	8.83 (8.81)	0–34	4	8.97 (8.99)	0–34	– 0.09 (4.06)
Cognitive Impairment (Items 6, 7, and 8; score range 0–15)	1	3.60 (2.80)	0–11	4	3.25 (3.05)	0–11	– 0.38 (1.19)
Visual Impairment (Items 9 and 10; score range 0–10)	1	1.54 (2.50)	0–10	4	1.47 (2.41)	0–10	– 0.16 (1.20)
Mood (Items 13, 14, 15, 16, 17, and 18; score range 0–30)	1	7.46 (5.19)	0–16	4	7.00 (5.96)	0–24	– 0.52 (4.08)
Treatment Burden (Items 21, 22, 23, 24, and 26; score range 0–38)	9	7.81 (7.08)	0–25	13	7.00 (6.35)	0–25	– 0.17 (3.23)
Total score (0–152)	1	33.94 (24.56)	0–99.4		30.95 (23.45)	2.3–92.0	– 3.15 (8.68)

^a^Mean (SD) and range are presented for numerical variables. Frequency of responses (*n*, [%]) are presented for categorical variables

^b^To quantify change from baseline, the categorical response options were given a numerical score ranging from 0 to 6

Domains are based on a conceptual model because the small sample size did not allow for domains to be constructed using factor analysis

*cTTP* Congenital thrombotic thrombocytopenic purpura, *cTTP-PEQ* Congenital Thrombotic Thrombocytopenic Purpura–Patient Experience Questionnaire, *NC* Not calculated, *PRO* Patient-reported outcome, *SD* Standard deviation, *TTP* Thrombotic thrombocytopenic purpura

### Validity

Spearman’s rank correlation coefficients for the cTTP-PEQ domains and total score and the existing PRO measures are shown in Table 3. Convergent validity was confirmed with moderate-to-high correlations (*r* ≥ 0.4) for 12 of the 15 hypothesized relationships between pairs of domains or total scores (Table 3). However, weak-to-moderate correlations were observed for three relationships that were hypothesized to have moderate-to-high correlations: the cTTP-PEQ Visual Impairment domain and the NEI-VFQ-25 Near Vision scale at baseline (*r* = – 0.18) and Day 14 (*r* = – 0.35), the cTTP-PEQ Pain/Bruising domain and the MCMDM Bleeding Questionnaire Bruising Subscale score at baseline (*r* = 0.32), and the cTTP-PEQ Activity Limitation single-item domain and the WPAI-GH “impairment while working due to health” score at baseline (*r* = 0.38) and Day 14 (*r* = 0.30). Discriminant validity was confirmed for five of the seven hypothesized relationships with low correlations (*r* < 0.3; Table 3). However, moderate-to-high correlations were observed for three of the relationships that were hypothesized to have low correlations: the cTTP-PEQ Fatigue single-item domain and the NEI-FVQ-25 Distant Vision scale at Day 14 (*r* = – 0.50), the cTTP-PEQ Bruising/Pain domain and the NEI-FVQ-25 Near Vision and Distance Vision scales at baseline (*r* = – 0.42 and – 0.45, respectively) and Day 14 (*r* = – 0.45 and – 0.42, respectively), and the cTTP-PEQ Treatment Burden domain and the NEI-FVQ-25 General Vision and Distant Vision scales at Day 14 (*r* = – 0.43 and – 0.48, respectively).

**Table 3:** Convergent and Discriminant Correlations Between the cTTP-PEQ Domain Scores and Existing PRO Measures

	cTTP-PEQ Domain Score																	
	Fatigue		Activity Limitation		Headache		Pain/Bruising		Cognitive Impairment		Visual Impairment		Mood		Treatment Burden		Total	
	Day 1	Day 14	Day 1	Day 14	Day 1	Day 14	Day 1	Day 14	Day 1	Day 14	Day 1	Day 14	Day 1	Day 14	Day 1	Day 14	Day 1	Day 14
**PROMIS-29**																		
Average pain rating	0.23	0.35	0.58	0.55	0.51	0.20	0.59^a^	0.74^a^	0.43	0.42	0.16	0.12	0.37	0.28	0.41	0.38	0.48	0.54
Social Roles/Activities	– 0.60	– 0.62	– 0.47^a^	– 0.61^a^	– 0.44	– 0.44	– 0.37	– 0.49	– 0.55	– 0.50	– 0.30	– 0.18	– 0.45	– 0.38	– 0.46^a^	– 0.42^a^	– 0.61	– 0.60
Anxiety/Fear	0.53	0.34	0.62	0.42	0.45	0.56	0.50	0.50	0.64	0.62	0.28	0.28	0.81^a^	0.72^a^	0.60	0.49	0.72	0.65
Depression/Sadness	0.55	0.55	0.50	0.44	0.31	0.34	0.51	0.44	0.61	0.69	0.36	0.21	0.79^a^	0.64^a^	0.54	0.52	0.76	0.67
Fatigue	0.86^a^	0.79^a^	0.56	0.56	0.45	0.36	0.48	0.45	0.43	0.50	0.28	0.22	0.67	0.51	0.67	0.51	0.78	0.68
Pain Interference	0.31	0.41	0.48	0.56	0.58	0.26	0.68^a^	0.69^a^	0.60	0.53	0.39	0.16	0.53	0.47	0.59	0.38	0.66	0.59
Physical Function	– 0.35	– 0.32	– 0.57	– 0.73	– 0.33	– 0.49	– 0.52	– 0.50	– 0.33	– 0.44	– 0.38	– 0.41	– 0.42	– 0.56	– 0.32	– 0.48	– 0.52	– 0.59
Sleep Disturbance	0.57	0.33	0.38	0.41	0.19	0.43	0.34	0.36	0.19	0.30	0.24	– 0.02	0.24	0.31	0.47	0.41	0.46	0.44
**HIT-6 total score**	0.62	0.53	0.47	0.47	0.57^a^	0.68^a^	0.55	0.65	0.31	0.46	0.28	0.20	0.58	0.41	0.71	0.77	0.70	0.71
**MCMDM Bleeding Questionnaire Bruising Subscale: total score**	0.21	0.21	– 0.05^b^	0.09^b^	0.29^b^	– 0.08^b^	0.32^c^	0.53^a^	0.16^b^	0.22^b^	0.19	– 0.04	0.17^b^	0.18^b^	0.36	0.31	0.26	0.38
**PDQ-5: total score**	0.22	0.40	0.21	0.37	0.41	0.43	0.47	0.57	0.68^a^	0.83^a^	0.40	0.39	0.58	0.66	0.44	0.57	0.57	0.74
**NEI-VFQ-25**																		
General Vision	– 0.19^b^	– 0.38^b^	– 0.33	– 0.40	– 0.11	– 0.23	– 0.26^b^	– 0.35^b^	– 0.35	– 0.45	– 0.56^a^	– 0.53^a^	– 0.41	– 0.54	– 0.31^b^	– 0.43^d^	– 0.41	– 0.55
Near Vision	– 0.17^b^	– 0.25^b^	– 0.31	– 0.54	– 0.40	– 0.31	– 0.42^d^	– 0.45^d^	– 0.35	– 0.35	– 0.18^c^	– 0.35^c^	– 0.27	– 0.39	– 0.20^b^	– 0.22^b^	– 0.34	– 0.43
Distant Vision	– 0.28^b^	– 0.50^d^	– 0.38	– 0.53	– 0.34	– 0.37	– 0.45^d^	– 0.42^d^	– 0.35	– 0.42	– 0.44^a^	– 0.66^a^	– 0.43	– 0.41	– 0.19^b^	– 0.48^d^	– 0.44	– 0.56
**WPAI-GH**																		
Work time missed	– 0.04	– 0.31	0.08	– 0.01	0.25	– 0.25	– 0.18	– 0.23	0.03	– 0.25	– 0.15	0.03	– 0.15	0.04	– 0.34	0.31	– 0.22	– 0.20
Impairment while working	0.20	0.23	0.38^c^	0.30^c^	0.41	0.33	0.12	0.66	0.29	0.46	0.25	0.32	0.03	0.41	0.46^a^	0.88^a^	0.21	0.73
Overall work impairment	0.16	0.22	0.34	0.35	0.41	0.30	0.06	0.59	0.26	0.40	0.20	0.32	0.01	0.36	0.31	0.90	0.13	0.65
Activity impairment	0.51	0.61	0.63^a^	0.51^a^	0.41	0.39	0.38	0.48	0.33	0.44	0.27	0.34	0.43	0.48	0.54^a^	0.79^a^	0.53	0.68
**PGI-S**	0.47	0.46	0.40	0.63	0.24	0.38	0.47	0.60	0.40	0.45	0.55	0.60	0.50	0.54	0.58	0.65	0.63	0.70

^a^Convergent validity confirmed; ^b^Discriminant validity confirmed; ^c^Convergent validity not confirmed; ^d^Discriminant validity not confirmed

Correlation coefficient values ≤ 0.19 were considered “very low,” < 0.3 were considered “low,” between 0.3 and 0.49 were considered “moderate,” > 0.5 were considered “high,” and  ≥ 0.7 were considered “very high”

Domains are based on a conceptual model because the small sample size did not allow for domains to be constructed using factor analysis

*cTTP-PEQ* Congenital Thrombotic Thrombocytopenic Purpura–Patient Experience Questionnaire, *HIT-6* Headache Impact Test, *MCMDM* Condensed Molecular and Clinical Markers for the Diagnosis and Management, *NEI-VFQ-25* National Eye Institute Visual Function Questionnaire-25, *PDQ-5* Perceived Deficits Questionnaire–5 items, *PGI-S* Patient Global Impression of Severity, *PRO* Patient-reported outcome, *PROMIS-29* Patient-Reported Outcomes Measurement Information System-29 Profile, *WPAI-GH* Work Productivity and Activity Impairment Questionnaire

Very high correlations (≥ 0.70) and very low correlations (≤ 0.19) that were not previously hypothesized were observed for the cTTP-PEQ domains and total score and other PRO measures at baseline and Day 14. These additional observed correlations are detailed in Table 3.

Known-groups validity was evaluated by observing that the mean cTTP-PEQ scores generally increased with higher patient-reported impression of cTTP severity, as measured by categorized PGI-S score (normal vs. mild/moderate/severe), for most items and domains at baseline and Day 14. The difference in mean cTTP-PEQ score between patients in the two PGI-S groups was significant (*p* ≤ 0.05) for all items except Items 4, 5, 6, 11, 12, 14, 15, 16, 19, 22, 24, and 26 at baseline and for all items, except Items 5, 6, 8, 17, 22, 24, and 26 at Day 14. The difference in cTTP-PEQ score between the two PGI-S groups was significant for all domains at baseline, with the exception of the single-item Activity Limitation score (*p* = 0.07) and single-item Headache score (*p* = 0.15), and for all domains at Day 14.

### Reliability

Item-to-total correlations for the cTTP-PEQ ranged from 0.32 (Item 12) to 0.75 (Item 18) at baseline and from – 0.03 (Item 26) to 0.81 (Item 8) on Day 14. The domain scores correlating the most with the total score were the Pain/Bruising domain (*r* = 0.79), the Mood score (*r* = 0.87), and the Treatment Burden score (*r* = 0.89). Individual items forming the multi-component domains showed moderate-to-high item-to-item correlation at baseline (Table S4) and Day 14 (Table S5).

No pair of items from the cTTP-PEQ had correlation equal to or greater than 0.90 at baseline or Day 14, suggesting an absence of redundancy in the information gathered from any pair of items. Item-to-item correlations ranged from 0.05 to 0.63 (baseline) and 0.11 to 0.58 (Day 14) for the Pain/Bruising domain (Items 2, 3, 4, 5, 12), from 0.39 to 0.54 (baseline) and 0.53 to 0.68 (Day 14) for the Cognitive Impairment domain (Items 6, 7, 8), from 0.28 to 0.73 (baseline) and 0.50 to 0.79 (Day 14) for the Mood domain (Items 13 to 18), and from 0.22 to 0.86 (baseline) and 0.03 to 0.59 (Day 14) for the Treatment Burden domain (Items 21, 22, 23, 24, 26). Item-to-item correlation was 0.72 (baseline) and 0.74 (Day 14) for the Visual Impairment domain (Items 9 and 10) (Table S4 and Table S5). Cronbach’s alpha was 0.88 at baseline and 0.91 on Day 14.

Test–retest reliability is the assessment of the internal validity of a measure, it measures the consistency of results when the same test is repeated on the same sample at a different time. The measurement of a stable construct should remain stable over time. Test–retest reliability was assessed using ICCs (1,2) between two time points (Day 1 and Day 14) for subjects who reported no changes in health status between both assessments. The ICC ranged from 0.762 for the Mood domain to 0.943 for the Cognitive Impairment domain. The ICC for the cTTP-PEQ total score was 0.955 (Table 4).

**Table 4:** Test–Retest Reliability of the cTTP-PEQ Domain Scores

Domain	*n*	Intraclass Correlation
**Pain/Bruising**	24	0.849
**Cognitive Impairment**	24	0.943
**Visual Impairment**	24	0.913
**Mood**	24	0.762
**Treatment Burden**	18	0.896
**Total cTTP score**	24	0.955

Analysis includes participants who reported “no change” on PGI-C on Day 14. Treatment Burden items were completed only by those who received TTP treatment in the past 2 weeks

Domains are based on a conceptual model because the small sample size did not allow for domains to be constructed using factor analysis

*cTTP* Congenital thrombotic thrombocytopenic purpura, *cTTP-PEQ* Congenital Thrombotic Thrombocytopenic Purpura–Patient Experience Questionnaire, *PGI-C* Patient Global Impression of Change, *PRO* Patient-reported outcome, *TTP* Thrombotic thrombocytopenic purpura

## Discussion

This study reports on the validation of a novel, 26-item, disease-specific PRO instrument for assessing the symptoms, impacts, and treatment-related considerations in patients with cTTP. The cTTP-PEQ was developed on the basis of the results of a literature review and concept elicitation interviews with clinicians and patients [12], followed by cognitive debriefing interviews with patients with cTTP. Validation of the cTTP-PEQ in 36 patients provides evidence of internal and test–retest reliability and of convergent, discriminant, and known-groups validity.

When evaluating the descriptive statistics for the cTTP-PEQ, floor effects were observed for the scores for individual items or hypothesized domains. The majority of patients scored at the bottom end of the possible response options available, resulting in lower variance. A possible explanation for the observed floor effects is that patients were not experiencing symptoms or impacts that were severe enough for them to score the items at the higher level. This is not unexpected, as most patients reported their severity level to be either normal (40.6%) or mild (37.5%). Items with high floor effects (> 40%) included those measuring confusion (Item 7), blurry vision (Item 9), anxiety (Item 17), experience of mood swings (Item 18), activity limitation (Item 19), and worry due to problems relating to TTP treatment (Item 21). A direct consequence of a high floor effect is that such items under-discriminate and, as a result, are potentially not sensitive to change [33]. It was not possible to assess how sensitive the cTTP-PEQ is to change during this study because of the small sample of patients reporting change.

Owing to the relatively small sample size, performing a factor analysis to assess the structural validity of the cTTP-PEQ was not possible. Originally, exploratory and confirmatory factor analysis was intended to be performed to investigate structural validity; however, this was not possible due to the nature and size of the sample distribution. The structure and domains of the proposed cTTP-PEQ instrument are based on a hypothetical conceptual model and framework, as well as item-to-item and item-to-total correlations. The cTTP-PEQ Pain/Bruising domain had the lowest support for its structural validity, with item-to-item correlations ranging from 0.05 to 0.63 at baseline and 0.11 to 0.58 on Day 14 and with moderate item-to-total correlations. This domain measures the worst pain experienced by the patient in the joints, muscle, abdomen, chest, and head in the past 24 h, and the worst bruising in the last 7 days. These low inter-item correlations may be explained by differences in the extent of thrombotic events at different locations in the body. Given certain domains have more items than others, and some items have a wider range of answer scores than others, the total score of the cTTP-PEQ could reflect a higher relative importance of domains with more items or items with higher possible scores. A factor analysis would have enabled a weighted sum of the measure, regardless of item numbers and score ranges per domain, but unfortunately the sample size did not permit these analyses.

Convergent and discriminant validity of the cTTP-PEQ was confirmed by the observed correlations between domains. Moderate-to-high correlations were observed between the domains of the cTTP-PEQ and related concepts of the alternative PRO measures in the majority of hypothesized cases. However, there were three hypothesized relationships for which convergent validity was not supported: the cTTP-PEQ Visual Impairment domain and NEI-VFQ-25 Near Vision scale, the cTTP-PEQ Pain/Bruising domain and the MCMDM Bleeding Questionnaire Bruising Subscale score at baseline, and the cTTP-PEQ Activity Limitation single-item domain and the WPAI-GH “impairment while working due to health” score. The MCMDM Bleeding Questionnaire Bruising Subscale focuses on bruising, whereas the cTTP-PEQ Pain/Bruising domain focuses largely on pain. Similarly, the cTTP-PEQ Visual Impairment domain measures blurry vision and blind spots, whereas the Near Vision scale of the NEI-VFQ-25 focuses on loss of near vision. This conceptual reasoning could go some way to explaining the observed weaker correlation. A similar explanation can be found for the cTTP-PEQ Activity Limitation domain, which focuses on activity limitation in a general sense, correlating poorly with the WPAI-GH, which focuses on productivity loss and inability to participate in work-related activities.

Test–retest reliability was assessed using ICCs (1,2) between two time points (Day 1 and Day 14) for subjects who reported no changes in health status between both assessments. While this is a common practice, it is potentially biased in that respondents self-select into the analysis set. Further, it ignores any measurement error associated with the anchor (health status change report). Low correlations between the domain and total scores measuring unrelated concepts were confirmed for most hypotheses. The exceptions were the moderate correlations observed between the cTTP-PEQ Fatigue single-item domain and NEI-FVQ-25 Distant Vision scale, the cTTP-PEQ Bruising/Pain domain and NEI-FVQ-25 Near and Distant Vision scales, and the cTTP-PEQ Treatment Burden domain and NEI-FVQ-25 General Vision and Distant Vision scales. The extent to which the hypotheses were confirmed generally supports the convergent and discriminant validity of the cTTP-specific instrument.

Known-groups validity was assessed by comparing the mean scores of the cTTP-PEQ between patients separated on the basis of their PGI-S score. The differences in the cTTP-PEQ scores between the PGI-S normal and mild/moderate/severe groups were statistically significant (*p* ≤ 0.05) for all domains and the total score except the Activity Limitation and Headache domains at baseline. Single Items 4, 5, 6, 11, 12, 14, 15, 16, 19, 22, 24, and 26 at baseline and Items 5, 6, 8, 17, 22, 24, and 26 at Day 14, also did not support known-groups validity. These findings can be linked to the high floor effects described earlier and the generally low levels of patient-reported cTTP severity, as measured by the PGI-S. Therefore, known-groups validity was partially supported by the results. At the score level however, the mean cTTP-specific instrument scores for most domains, as well as the total score, increased with the increase of the patient-reported impression of cTTP severity, as measured by the PGI-S, and this difference was statistically significant, supporting known-groups validity (Table S6).

The Cronbach’s alpha values calculated for the cTTP-PEQ indicate that the measure has a high degree of internal consistency. In addition, the high ICC values calculated for patients who reported no changes in health status between baseline and Day 14 indicate that the test has high test–retest reliability.

A major limitation of this study was the difficulty recruiting patients with this ultra-rare disease. A range of solutions were implemented to maximize recruitment; however, the enrolled patient population represents a convenience sample of patients whose disease may be mild or adequately controlled owing to prophylactic treatment. Due to high mortality rates associated with severe cTTP, the majority of patients (77.8%) were receiving prophylactic treatment and did not modify their treatment during the course of the study. The control of cTTP symptoms by treatment may explain why most clinician- and patient-reported disease severity levels were mild. As such, patient responses were on the lower end of the scoring range, contributing to the aforementioned floor effects observed in this study. In addition, the small sample size prevented more robust assessment of the psychometric properties from being performed, and structural validity could not be assessed using a factor analysis. Therefore, item-to-item and item-to-total correlations were used to support the grouping of items, following the hypothesized conceptual model. In addition, a pragmatic scoring method was used to summarize individual items into domain scores. Therefore, the response options and number of items per domain will weight items differently using this approach. Considering the small sample size, it should also be noted that many of the bivariate correlation findings should be interpreted with caution, especially for the single items. The limited sample size also affects the feasibility of assessing the validity of single items as estimators. Future studies could aim to assess the cTTP-PEQ in patients with more severe disease to provide further insight on whether the observed floor effects are inherent to the sample or the PRO measure, and could allow the assessment of sensitivity to change. However, patients with severe disease are uncommon in the clinical environment due to the use of prophylactic treatment and would be challenging to recruit. Ideally, modern test theory methods would be used to further examine the alignment of the response options with patient severities and assess replicability of the findings. However, these methods would require larger sample sizes.

## Conclusions

Patients with the ADAMTS13 deficiency cTTP experience disease- and treatment-related complications and burdens that have a negative impact on their HRQoL [9, 10]. Although several generic PRO instruments have been used to investigate the impact of TTP and associated treatment on patients’ HRQoL, these instruments have never fully addressed the experience of patients with cTTP [19, 20]. The findings of this study support the validity and reliability of a cTTP-specific PRO instrument to assess the disease- and treatment-related burden of cTTP. This is the first disease-specific instrument to be implemented in patients with cTTP and this study represents an important step towards capturing and quantifying the disease and treatment experience of patients with this ultra-rare disorder. This instrument has the potential to aid our understanding, measurement, and interpretation of the effect of new treatments and how they can improve patients’ HRQoL.

## Electronic supplementary material

Below is the link to the electronic supplementary material.

Supplementary Material 1

## Data Availability

The datasets, including the redacted study protocol, redacted statistical analysis plan, and individual participants data supporting the results reported in this article, will be made available within 3 months from initial request to researchers who provide a methodologically sound proposal. The data will be provided after its de-identification, in compliance with applicable privacy laws, data protection, and requirements for consent and anonymization.

## Abbreviations

A36 Hemofilia-QoL: Haemophilia Quality of Life Questionnaire for Adults
ADAMTS13: A disintegrin and metalloproteinase with a thrombospondin type 1 motif member 13
cTTP: Congenital thrombotic thrombocytopenic purpura
cTTP-PEQ: Congenital Thrombotic Thrombocytopenic Purpura–Patient Experience Questionnaire
FVIII: Factor VIII
Haemo-QoL: Haemophilia Quality of Life Questionnaire for Children
HIT-6: Headache Impact Test
HRQoL: Health-related quality of life
ICC: Intraclass correlation coefficient
MCMDM: Condensed Molecular and Clinical Markers for the Diagnosis and Management
NEI-VFQ-25: National Eye Institute Visual Function Questionnaire-25
PDQ-5: Perceived Deficits Questionnaire–5 items
PGI-C: Patient Global Impression of Change
PGI-S: Patient Global Impression of Severity
PRO: Patient-reported outcome
PROMIS-29: Patient-Reported Outcomes Measurement Information System-29 Profile
TTP: Thrombotic thrombocytopenic purpura
VWF: von Willebrand factor
WPAI-GH: Work Productivity and Activity Impairment Questionnaire
