# Supplementary material for: Psychometric evaluation of a patient-reported outcomes instrument for congenital thrombotic thrombocytopenic purpura
Source: J Patient Rep Outcomes. 2023 Jul 14;7:68. doi: 10.1186/s41687-023-00592-w (PMC10349015; doi:10.1186/s41687-023-00592-w)
Supplement: Supplementary file 1 — Supplementary Material 1 [file 41687_2023_592_MOESM1_ESM.docx]

# SUPPLEMENTARY TABLES

**Table S1** Hypothesized cTTP-PEQ Items, Domains, and Scoring Range

| cTTP-Specific PRO Instrument Item or Domain | Score Range |
| --- | --- |
| **Item 1. During the past 24 hours, how bad was your worst fatigue?** | 0–10 |
| **Item 2. During the past 24 hours, how bad was your worst joint pain?** | 0–10 |
| **Item 3. During the past 24 hours, how bad was your worst muscle pain?** | 0–10 |
| **Item 4. During the past 24 hours, how bad was your worst abdominal pain?** | 0–10 |
| **Item 5. During the past 24 hours, how bad was your worst chest pain?** | 0–10 |
| **Item 6. During the past 7 days, how often were you forgetful?** | 0–5 |
| None of the time |  |
| A little of the time |  |
| Some of the time |  |
| A good bit of the time |  |
| Most of the time |  |
| All of the time |  |
| **Item 7. During the past 7 days, how often were you confused?** | 0–5 |
| None of the time |  |
| A little of the time |  |
| Some of the time |  |
| A good bit of the time |  |
| Most of the time |  |
| All of the time |  |
| **Item 8. During the past 7 days, how often did you have difficulty finding the right words to say during conversations?** | 0–5 |
| None of the time |  |
| A little of the time |  |
| Some of the time |  |
| A good bit of the time |  |
| Most of the time |  |
| All of the time |  |
| **Item 9. During the past 7 days, how often did you have blurry vision?** | 0–5 |
| None of the time |  |
| A little of the time |  |
| Some of the time |  |
| A good bit of the time |  |
| Most of the time |  |
| All of the time |  |
| **Item 10. During the past 7 days, how often did you experience blind spots?** | 0–5 |
| None of the time |  |
| A little of the time |  |
| Some of the time |  |
| A good bit of the time |  |
| Most of the time |  |
| All of the time |  |
| **Item 11. During the past 7 days, how often did you have a headache (including migraines)?** | 0–5 |
| None of the time |  |
| A little of the time |  |
| Some of the time |  |
| A good bit of the time |  |
| Most of the time |  |
| All of the time |  |
| **Item 12. During the past 7 days, how bad was your worst bruising?** | 0–5 |
| **Item 13. During the past 7 days, how often did you feel depressed?** | 0–5 |
| None of the time |  |
| A little of the time |  |
| Some of the time |  |
| A good bit of the time |  |
| Most of the time |  |
| All of the time |  |
| **Item 14. During the past 7 days, how often did you feel angry?** | 0–5 |
| None of the time |  |
| A little of the time |  |
| Some of the time |  |
| A good bit of the time |  |
| Most of the time |  |
| All of the time |  |
| **Item 15. During the past 7 days, how often did you feel irritable?** | 0–5 |
| None of the time |  |
| A little of the time |  |
| Some of the time |  |
| A good bit of the time |  |
| Most of the time |  |
| All of the time |  |
| **Item 16. During the past 7 days, how often did you feel frustrated?** | 0–5 |
| None of the time |  |
| A little of the time |  |
| Some of the time |  |
| A good bit of the time |  |
| Most of the time |  |
| All of the time |  |
| **Item 17. During the past 7 days, how often did you feel anxious?** | 0–5 |
| None of the time |  |
| A little of the time |  |
| Some of the time |  |
| A good bit of the time |  |
| Most of the time |  |
| All of the time |  |
| **Item 18. During the past 7 days, how often did you experience mood swings?** | 0–5 |
| None of the time |  |
| A little of the time |  |
| Some of the time |  |
| A good bit of the time |  |
| Most of the time |  |
| All of the time |  |
| **Item 19. During the past 7 days, how much were your daily activities limited?** | 0–4 |
| Not at all |  |
| A little bit |  |
| Moderately |  |
| Quite a bit |  |
| Extremely |  |
| **Item 20. During the past 2 weeks, did you receive treatment for TTP?** |  |
| No | 0 |
| Yes | 1 |
| **Item 21. During the past 2 weeks, how much did you worry there might be problems resulting from your treatment for TTP?** | 0–4 |
| Not at all |  |
| A little bit |  |
| Moderately |  |
| Quite a bit |  |
| Extremely |  |
| **Item 22. During the past 2 weeks, how bad was your allergic reaction to your treatment for TTP?** | 0–10 |
| **Item 23. During the past 2 weeks, how bad was your drowsiness related to your treatment for TTP?** | 0–10 |
| **Item 24. During the past 2 weeks, how bad was your pain due to injection, IV insertion, or port access for your TTP treatment?** | 0–10 |
| **Item 25a. Check here if you did not miss any work/school** | 0 |
| No |  |
| Yes |  |
| **Item 25b. Check here if you do not work outside of the home or attend school** | 0 |
| No |  |
| Yes |  |
| **Item 25c. During the past 2 weeks, how many hours of work/school did you miss because of your treatment for TTP?** | Number of hours |
| **Item 26. During the past 2 weeks, how much did the travel to receive your treatment for TTP feel like a burden (i.e., the actual distance or getting someone to go with you)?** | 0–4 |
| Not at all |  |
| A little bit |  |
| Moderately |  |
| Quite a bit |  |
| Extremely |  |
| **Domain score** |  |
| Fatigue (Item 1) | 0–10 |
| Headache (Item 11) | 0–5 |
| Activity Limitations (Item 19) | 0–4 |
| Pain/Bruising (Items 2, 3, 4, 5, and 12) | 0–45 |
| Cognitive Impairment (Items 6, 7, and 8) | 0–15 |
| Visual Impairment (Items 9 and 10) | 0–10 |
| Mood (Items 13, 14, 15, 16, 17, and 18) | 0–30 |
| Treatment Burden (Items 21, 22, 23, 24, and 26) | 0–38 |
| Total score | 0–152 |

Items 20 and 25 were not included in the domain scoring. If TTP treatment was received (Item 20), questions 21–26 were completed

Domains are based on a conceptual model because the small sample size did not allow for domains to be constructed using factor analysis

*cTTP* Congenital thrombotic thrombocytopenic purpura; *IV* Intravenous, *TTP* Thrombotic thrombocytopenic purpura

**Table S2** Hypothesized Relationships Between the cTTP-PEQ Domains and Existing PRO Instrument Constructs

| **Hypothesized moderate to high correlations to assess convergent validity** |
| --- |
| - cTTP Fatigue domain and PROMIS-29 Fatigue - cTTP Headache domain and HIT-6 total score - cTTP Pain/Bruising domain and PROMIS-29 Pain Interference, PROMIS-29 Pain Intensity, and MCMDM Bleeding Questionnaire Bruising Subscale total score - cTTP Cognitive Impairment domain and PDQ-5 - cTTP Visual Impairment domain and NEI-VFQ-25 - cTTP Mood domain with PROMIS-29 Depression/Sadness and PROMIS-29 Anxiety/Fear - cTTP Activity Limitation domain with PROMIS-29 Ability to participate in social roles and activities, WPAI-GH Impairment while working, and WPAI-GH Activity impairment - cTTP Treatment Burden domain with PROMIS-29 Social Roles/Activities, WPAI-GH Impairment while working, and WPAI-GH Activity impairment |
| **Hypothesized low correlations to assess discriminant validity^a^** |
| - cTTP Fatigue domain and NEI-VFQ-25 - cTTP Activity Limitation domain and MCMDM Bleeding Questionnaire Bruising Subscale total score - cTTP Pain/Bruising domain and NEI-VFQ-25 - cTTP Headache domain and MCMDM Bleeding Questionnaire Bruising Subscale total score - cTTP Mood domain and MCMDM Bleeding Questionnaire Bruising Subscale total score - cTTP Cognitive Impairment domain and MCMDM Bleeding Questionnaire Bruising Subscale total score - cTTP Treatment Burden domain and NEI-VFQ-25 |

Correlation coefficient values ≤ 0.19 were considered “very low,” < 0.3 were considered “low,” between 0.3 and 0.49 were considered “moderate,” > 0.5 were considered “high,” and ≥ 0.7 were considered “very high”

^a^There are fewer hypothesized relationships among the PRO instruments for discriminant validity as many of the instruments were expected to be related

*cTTP* Congenital thrombotic thrombocytopenic purpura; *HIT-6* Headache Impact Test, *MCMDM* Condensed Molecular and Clinical Markers for the Diagnosis and Management, *NEI-VFQ-25* National Eye Institute Visual Function Questionnaire-25, *PDQ-5* Perceived Deficits Questionnaire – 5 items, *PRO* Patient-reported outcome, *PROMIS-29* Patient-Reported Outcomes Measurement Information System-29 Profile; *WPAI-GH* Work Productivity and Activity Impairment Questionnaire

**Table S3** Descriptive Statistics for Other PRO Instrument Domains and Total Score

| PRO Instrument Domain | *N* = 36 | | | | | | Change from Baseline (SD)^b^ |
| --- | --- | --- | --- | --- | --- | --- | --- |
|  | Baseline | | | Day 14 | | |  |
|  | Missing, *n* | Mean (SD) or *n* (%)^a^ | Range | Missing, *n* | Mean (SD) or *n* (%)^a^ | Range |  |
| **PROMIS-29 [25]** |  |  |  |  |  |  |  |
| Social Roles/Activities | 4 | 52.26 (9.31) | 32.0–64.2 | 4 | 55.16 (10.44) | 32.0–64.2 | 3.16 (5.19) |
| Anxiety/Fear | 4 | 51.58 (11.48) | 40.3–75.4 | 4 | 51.37 (11.52) | 40.3–75.8 | – 0.11 (6.50) |
| Depression/Sadness | 4 | 50.15 (10.44) | 41.0–79.3 | 4 | 50.42 (9.37) | 41.0–79.3 | 0.07 (6.61) |
| Fatigue | 4 | 52.30 (11.52) | 33.7–75.8 | 4 | 51.28 (10.29) | 33.7–69.0 | – 0.99 (6.23) |
| Pain Interference | 4 | 48.94 (9.52) | 41.6–75.6 | 4 | 48.09 (8.75) | 41.6–66.7 | – 0.57 (5.96) |
| Physical Function | 4 | 51.89 (8.92) | 32.1–57.0 | 4 | 51.18 (8.73) | 32.1–57.0 | – 0.75 (4.28) |
| Sleep Disturbance | 4 | 11.31 (3.71) | 6.0–19.0 | 4 | 11.13 (4.49) | 4.0–20.0 | – 0.27 (2.60) |
| **HIT-6 total score [26]** | 4 | 52.59 (8.92) | 36–66 | 6 | 50.83 (10.54) | 36–74 | – 1.79 (5.31) |
| **MCMDM Bleeding Questionnaire Bruising Subscale [27]** |  |  |  |  |  |  |  |
| Total Bruising | 4 | 0.31 (0.54) | 0–2 | 6 | 0.33 (0.48) | 0–1 | 0.00 (0.47) |
| **PDQ-5 total score [28]** | 4 | 7.56 (5.47) | 0–19 | 6 | 7.41 (5.33) | 0–19 | – 0.27 (2.55) |
| **NEI-VFQ-25 [29]** |  |  |  |  |  |  |  |
| Near Vision | 4 | 88.02 (19.62) | 16.7–100.0 | 4 | 88.54 (18.42) | 16.7–100.0 | 0.56 (7.56) |
| Distant Vision | 4 | 86.98 (18.69) | 25.0–100.0 | 4 | 88.67 (19.58) | 16.7–100.0 | 1.81 (9.26) |
| **WPAI-GH [30]** |  |  |  |  |  |  |  |
| Percentage work time missed due to health | 20 | 6.71 (16.59) | 0.0–56.5 | 21 | 13.31 (26.78) | 0.0–100.0 | 8.11 (33.70) |
| Percentage impairment while working due to health | 21 | 15.33 (19.59) | 0.0–60.0 | 23 | 27.69 (32.70) | 0.0–80.0 | 8.00 (21.50) |
| Percentage overall work impairment due to health | 21 | 19.28 (26.28) | 0.0–76.0 | 23 | 31.10 (34.30) | 0.0–80.0 | 6.92 (23.01) |
| Percentage activity impairment due to health | 6 | 31.00 (32.09) | 0.0–100.0 | 6 | 31.00 (30.44) | 0.0–90.0 | – 0.71 (17.83) |

^a^Mean (SD) and range are presented for numerical variables. Frequency of responses (*n*, [%]) are presented for categorical variables

^b^To quantify change from baseline, the categorical response options were given a numerical score ranging from 0 to 100 depending on the measure

*HIT-6* Headache Impact Test, *MCMDM* Condensed Molecular and Clinical Markers for the Diagnosis and Management, *NEI-VFQ-25* National Eye Institute Visual Function Questionnaire-25, *PDQ-5* Perceived Deficits Questionnaire – 5 items, *PRO* Patient-reported outcome, *PROMIS-29* Patient-Reported Outcomes Measurement Information System-29 Profile; *SD* Standard deviation, *WPAI-GH* Work Productivity and Activity Impairment Questionnaire

**Table S4** Item-to-Item Correlations for the cTTP-PEQ at Baseline

| Item | 1 | 2 | 3 | 4 | 5 | 6 | 7 | 8 | 9 | 10 | 11 | 12 | 13 | 14 | 15 | 16 | 17 | 18 | 19 | 21 | 22 | 23 | 24 | 26 |
| --- | --- | --- | --- | --- | --- | --- | --- | --- | --- | --- | --- | --- | --- | --- | --- | --- | --- | --- | --- | --- | --- | --- | --- | --- |
| 1 | 1.00 |  |  |  |  |  |  |  |  |  |  |  |  |  |  |  |  |  |  |  |  |  |  |  |
| 2 | 0.32 | 1.00 |  |  |  |  |  |  |  |  |  |  |  |  |  |  |  |  |  |  |  |  |  |  |
| 3 | 0.46 | 0.47 | 1.00 |  |  |  |  |  |  |  |  |  |  |  |  |  |  |  |  |  |  |  |  |  |
| 4 | 0.28 | 0.32 | 0.47 | 1.00 |  |  |  |  |  |  |  |  |  |  |  |  |  |  |  |  |  |  |  |  |
| 5 | 0.33 | 0.46 | 0.53 | 0.63 | 1.00 |  |  |  |  |  |  |  |  |  |  |  |  |  |  |  |  |  |  |  |
| 6 | 0.37 | 0.33 | 0.23 | 0.11 | 0.25 | 1.00 |  |  |  |  |  |  |  |  |  |  |  |  |  |  |  |  |  |  |
| 7 | 0.43 | 0.37 | 0.48 | 0.40 | 0.43 | 0.54 | 1.00 |  |  |  |  |  |  |  |  |  |  |  |  |  |  |  |  |  |
| 8 | 0.19 | 0.40 | 0.40 | 0.17 | 0.14 | 0.41 | 0.39 | 1.00 |  |  |  |  |  |  |  |  |  |  |  |  |  |  |  |  |
| 9 | 0.26 | 0.20 | 0.33 | 0.40 | 0.32 | 0.24 | 0.40 | 0.28 | 1.00 |  |  |  |  |  |  |  |  |  |  |  |  |  |  |  |
| 10 | 0.12 | 0.13 | 0.03 | 0.41 | 0.23 | 0.10 | 0.20 | 0.16 | 0.72 | 1.00 |  |  |  |  |  |  |  |  |  |  |  |  |  |  |
| 11 | 0.34 | 0.12 | 0.44 | 0.26 | 0.19 | 0.09 | 0.29 | 0.32 | 0.03 | – 0.12 | 1.00 |  |  |  |  |  |  |  |  |  |  |  |  |  |
| 12 | 0.09 | 0.27 | 0.05 | 0.07 | 0.06 | 0.12 | 0.35 | 0.03 | 0.22 | 0.19 | 0.39 | 1.00 |  |  |  |  |  |  |  |  |  |  |  |  |
| 13 | 0.44 | 0.44 | 0.38 | 0.19 | 0.25 | 0.56 | 0.62 | 0.20 | 0.33 | 0.20 | 0.06 | 0.32 | 1.00 |  |  |  |  |  |  |  |  |  |  |  |
| 14 | 0.44 | 0.38 | 0.35 | 0.29 | 0.46 | 0.34 | 0.57 | 0.33 | 0.30 | 0.26 | 0.25 | 0.24 | 0.49 | 1.00 |  |  |  |  |  |  |  |  |  |  |
| 15 | 0.44 | 0.22 | 0.26 | 0.33 | 0.34 | 0.05 | 0.39 | 0.28 | 0.30 | 0.32 | 0.19 | 0.13 | 0.28 | 0.71 | 1.00 |  |  |  |  |  |  |  |  |  |
| 16 | 0.40 | 0.45 | 0.46 | 0.33 | 0.35 | 0.14 | 0.36 | 0.62 | 0.19 | 0.17 | 0.40 | 0.11 | 0.36 | 0.50 | 0.64 | 1.00 |  |  |  |  |  |  |  |  |
| 17 | 0.54 | 0.29 | 0.45 | 0.16 | 0.16 | 0.55 | 0.59 | 0.43 | 0.31 | 0.20 | 0.26 | 0.24 | 0.73 | 0.39 | 0.40 | 0.47 | 1.00 |  |  |  |  |  |  |  |
| 18 | 0.58 | 0.40 | 0.50 | 0.43 | 0.36 | 0.50 | 0.59 | 0.32 | 0.26 | 0.16 | 0.32 | 0.22 | 0.61 | 0.65 | 0.54 | 0.48 | 0.72 | 1.00 |  |  |  |  |  |  |
| 19 | 0.53 | 0.46 | 0.41 | 0.29 | 0.26 | 0.14 | 0.23 | 0.25 | 0.12 | – 0.03 | 0.57 | 0.24 | 0.21 | 0.33 | 0.34 | 0.48 | 0.41 | 0.50 | 1.00 |  |  |  |  |  |
| 21 | 0.26 | 0.50 | 0.38 | 0.16 | 0.11 | 0.05 | 0.15 | 0.32 | 0.45 | 0.60 | 0.08 | 0.17 | 0.25 | 0.37 | 0.46 | 0.25 | 0.32 | 0.38 | 0.21 | 1.00 |  |  |  |  |
| 22 | 0.32 | 0.44 | 0.39 | 0.28 | 0.14 | 0.01 | 0.07 | 0.27 | 0.47 | 0.57 | 0.02 | 0.08 | 0.22 | 0.23 | 0.29 | 0.20 | 0.28 | 0.36 | 0.35 | 0.86 | 1.00 |  |  |  |
| 23 | 0.54 | 0.36 | 0.50 | 0.53 | 0.32 | 0.41 | 0.45 | 0.50 | 0.48 | 0.54 | 0.11 | – 0.12 | 0.35 | 0.28 | 0.51 | 0.41 | 0.56 | 0.57 | 0.31 | 0.58 | 0.55 | 1.00 |  |  |
| 24 | 0.46 | 0.08 | 0.05 | 0.13 | 0.26 | 0.30 | 0.29 | 0.17 | 0.30 | 0.42 | 0.37 | 0.45 | 0.27 | 0.39 | 0.51 | 0.24 | 0.41 | 0.36 | 0.22 | 0.22 | 0.25 | 0.37 | 1.00 |  |
| 26 | 0.31 | 0.27 | 0.10 | 0.26 | 0.24 | 0.26 | – 0.20 | 0.01 | 0.21 | 0.29 | – 0.27 | – 0.12 | 0.01 | – 0.09 | 0.13 | – 0.07 | 0.11 | 0.17 | 0.07 | 0.32 | 0.39 | 0.40 | 0.25 | 1.00 |

This analysis is exploratory given the lack of variability found in the descriptive analysis results. Items 20 and 25 were excluded from total scoring and psychometric analyses and so are not presented in the table

*cTTP* Congenital thrombotic thrombocytopenic purpura, *PRO* Patient-reported outcome

**Table S5** Item-to-Item Correlations for the cTTP-PEQ at Day 14

| Item | 1 | 2 | 3 | 4 | 5 | 6 | 7 | 8 | 9 | 10 | 11 | 12 | 13 | 14 | 15 | 16 | 17 | 18 | 19 | 21 | 22 | 23 | 24 | 26 |
| --- | --- | --- | --- | --- | --- | --- | --- | --- | --- | --- | --- | --- | --- | --- | --- | --- | --- | --- | --- | --- | --- | --- | --- | --- |
| 1 | 1.00 |  |  |  |  |  |  |  |  |  |  |  |  |  |  |  |  |  |  |  |  |  |  |  |
| 2 | 0.36 | 1.00 |  |  |  |  |  |  |  |  |  |  |  |  |  |  |  |  |  |  |  |  |  |  |
| 3 | 0.46 | 0.58 | 1.00 |  |  |  |  |  |  |  |  |  |  |  |  |  |  |  |  |  |  |  |  |  |
| 4 | 0.31 | 0.35 | 0.44 | 1.00 |  |  |  |  |  |  |  |  |  |  |  |  |  |  |  |  |  |  |  |  |
| 5 | 0.29 | 0.26 | 0.47 | 0.54 | 1.00 |  |  |  |  |  |  |  |  |  |  |  |  |  |  |  |  |  |  |  |
| 6 | 0.56 | 0.44 | 0.36 | 0.41 | 0.25 | 1.00 |  |  |  |  |  |  |  |  |  |  |  |  |  |  |  |  |  |  |
| 7 | 0.37 | 0.34 | 0.42 | 0.50 | 0.41 | 0.67 | 1.00 |  |  |  |  |  |  |  |  |  |  |  |  |  |  |  |  |  |
| 8 | 0.24 | 0.36 | 0.34 | 0.30 | 0.36 | 0.68 | 0.53 | 1.00 |  |  |  |  |  |  |  |  |  |  |  |  |  |  |  |  |
| 9 | 0.18 | 0.10 | 0.24 | 0.32 | 0.49 | 0.30 | 0.43 | 0.43 | 1.00 |  |  |  |  |  |  |  |  |  |  |  |  |  |  |  |
| 10 | 0.11 | 0.11 | 0.00 | 0.24 | 0.33 | 0.30 | 0.43 | 0.51 | 0.74 | 1.00 |  |  |  |  |  |  |  |  |  |  |  |  |  |  |
| 11 | 0.24 | 0.20 | 0.58 | 0.24 | 0.34 | 0.19 | 0.20 | 0.30 | 0.19 | 0.13 | 1.00 |  |  |  |  |  |  |  |  |  |  |  |  |  |
| 12 | 0.35 | 0.56 | 0.30 | 0.24 | 0.11 | 0.25 | 0.43 | 0.14 | 0.10 | 0.25 | 0.13 | 1.00 |  |  |  |  |  |  |  |  |  |  |  |  |
| 13 | 0.42 | 0.34 | 0.28 | 0.45 | 0.40 | 0.65 | 0.51 | 0.35 | 0.35 | 0.39 | – 0.07 | 0.26 | 1.00 |  |  |  |  |  |  |  |  |  |  |  |
| 14 | 0.36 | 0.08 | 0.32 | 0.49 | 0.14 | 0.58 | 0.72 | 0.38 | 0.32 | 0.33 | 0.18 | 0.23 | 0.52 | 1.00 |  |  |  |  |  |  |  |  |  |  |
| 15 | 0.41 | 0.45 | 0.57 | 0.47 | 0.39 | 0.54 | 0.49 | 0.46 | 0.24 | 0.38 | 0.46 | 0.34 | 0.56 | 0.66 | 1.00 |  |  |  |  |  |  |  |  |  |
| 16 | 0.21 | 0.23 | 0.25 | 0.67 | 0.44 | 0.61 | 0.76 | 0.58 | 0.39 | 0.56 | 0.31 | 0.36 | 0.56 | 0.78 | 0.66 | 1.00 |  |  |  |  |  |  |  |  |
| 17 | 0.35 | 0.28 | 0.48 | 0.53 | 0.33 | 0.56 | 0.60 | 0.45 | 0.28 | 0.21 | 0.35 | 0.14 | 0.51 | 0.52 | 0.56 | 0.51 | 1.00 |  |  |  |  |  |  |  |
| 18 | 0.15 | 0.31 | 0.50 | 0.46 | 0.39 | 0.45 | 0.54 | 0.47 | 0.37 | 0.36 | 0.22 | 0.23 | 0.60 | 0.68 | 0.79 | 0.68 | 0.50 | 1.00 |  |  |  |  |  |  |
| 19 | 0.31 | 0.39 | 0.61 | 0.42 | 0.52 | 0.32 | 0.34 | 0.30 | 0.58 | 0.30 | 0.41 | 0.14 | 0.25 | 0.25 | 0.37 | 0.30 | 0.33 | 0.37 | 1.00 |  |  |  |  |  |
| 21 | 0.46 | 0.70 | 0.57 | 0.32 | 0.58 | 0.61 | 0.56 | 0.69 | 0.63 | 0.64 | 0.34 | 0.57 | 0.41 | 0.38 | 0.68 | 0.48 | 0.42 | 0.55 | 0.65 | 1.00 |  |  |  |  |
| 22 | – 0.02 | 0.19 | – 0.05 | 0.13 | 0.02 | 0.26 | 0.37 | 0.38 | 0.48 | 0.67 | 0.06 | 0.33 | 0.27 | 0.56 | 0.39 | 0.46 | 0.27 | 0.29 | 0.04 | 0.47 | 1.00 |  |  |  |
| 23 | 0.19 | 0.21 | 0.36 | 0.31 | 0.19 | 0.41 | 0.60 | 0.59 | 0.57 | 0.71 | 0.44 | 0.20 | 0.35 | 0.61 | 0.52 | 0.60 | 0.53 | 0.46 | 0.35 | 0.48 | 0.59 | 1.00 |  |  |
| 24 | 0.38 | 0.54 | 0.24 | – 0.09 | 0.09 | 0.54 | 0.26 | 0.47 | 0.29 | 0.30 | 0.08 | 0.57 | 0.43 | 0.23 | 0.45 | 0.21 | 0.22 | 0.26 | 0.16 | 0.52 | 0.37 | 0.09 | 1.00 |  |
| 26 | – 0.26 | – 0.18 | – 0.08 | 0.07 | – 0.23 | – 0.09 | – 0.14 | 0.00 | – 0.09 | – 0.05 | – 0.19 | – 0.08 | – 0.23 | 0.23 | 0.06 | – 0.02 | – 0.23 | 0.17 | – 0.08 | 0.08 | 0.21 | 0.03 | 0.04 | 1.00 |

This analysis is exploratory given the lack of variability found in the descriptive analysis results. Items 20 and 25 were excluded from total scoring and psychometric analyses and so are not presented in the table

*cTTP* Congenital thrombotic thrombocytopenic purpura, *PRO* Patient-reported outcome

**Table S6** Known-groups Validity: cTTP-specific Instrument Domain and Total Score by Patient Global Impression of Severity (PGIS) grouped in normal vs mild/moderate/severe

|  |  | **Day 1** | | | **Day 14** | | |  |
| --- | --- | --- | --- | --- | --- | --- | --- | --- |
|  | **PGIS Group** | **N** | **Mean (SD)** | **p-value** | **N** | **Mean (SD)** | **p-value** |  |
| **Individual item scores** |  |  |  |  |  |  |  |  |
| 1. During the past 24 hours, how bad was your worst fatigue? | Normal | 13 | 3.08 (2.96) | 0.04 | 17 | 2.65 (1.93) | 0.05 |  |
|  | Mild/Moderate/Severe | 19 | 5.37 (2.87) |  | 14 | 4.64 (3.15) |  |  |
| 2. During the past 24 hours, how bad was your worst joint pain? | Normal | 13 | 1.08 (1.85) | 0.02 | 18 | 1.17 (1.82) | 0.00 |  |
|  | Mild/Moderate/Severe | 19 | 3.00 (2.62) |  | 14 | 3.50 (2.31) |  |  |
| 3. During the past 24 hours, how bad was your worst muscle pain? | Normal | 13 | 0.69 (1.03) | 0.02 | 18 | 0.72 (0.96) | 0.01 |  |
|  | Mild/Moderate/Severe | 19 | 2.53 (3.06) |  | 14 | 3.14 (2.98) |  |  |
| 4. During the past 24 hours, how bad was your worst abdominal pain? | Normal | 13 | 0.62 (1.66) | 0.05 | 18 | 0.61 (1.09) | 0.03 |  |
|  | Mild/Moderate/Severe | 19 | 2.47 (3.41) |  | 14 | 2.93 (3.56) |  |  |
| 5. During the past 24 hours, how bad was your worst chest pain? | Normal | 13 | 0.46 (1.66) | 0.58 | 18 | 0.11 (0.32) | 0.08 |  |
|  | Mild/Moderate/Severe | 19 | 0.79 (1.58) |  | 14 | 1.07 (1.86) |  |  |
| 6. During the past 7 days, how often were you forgetful? | Normal | 13 | 1.31 (0.95) | 0.39 | 18 | 1.11 (1.02) | 0.07 |  |
|  | Mild/Moderate/Severe | 19 | 1.63 (1.16) |  | 14 | 2.00 (1.47) |  |  |
| 7. During the past 7 days, how often were you confused? | Normal | 13 | 0.15 (0.38) | 0.00 | 18 | 0.22 (0.43) | 0.01 |  |
|  | Mild/Moderate/Severe | 19 | 1.21 (1.23) |  | 14 | 1.29 (1.20) |  |  |
| 8. During the past 7 days, how often did you have difficulty finding the right words to say during conversations? | Normal | 13 | 0.77 (0.83) | 0.03 | 18 | 0.72 (0.89) | 0.06 |  |
|  | Mild/Moderate/Severe | 19 | 1.68 (1.42) |  | 14 | 1.50 (1.22) |  |  |
| 9. During the past 7 days, how often did you have blurry vision? | Normal | 13 | 0.31 (0.48) | 0.01 | 18 | 0.28 (0.57) | 0.01 |  |
|  | Mild/Moderate/Severe | 19 | 1.37 (1.37) |  | 14 | 1.64 (1.60) |  |  |
| 10. During the past 7 days, how often did you experience blind spots? | Normal | 13 | 0.23 (0.44) | 0.04 | 18 | 0.06 (0.24) | 0.02 |  |
|  | Mild/Moderate/Severe | 19 | 1.11 (1.63) |  | 14 | 1.29 (1.77) |  |  |
| 11. During the past 7 days, how often did you have a headache (including migraines)? | Normal | 13 | 0.85 (0.90) | 0.15 | 18 | 0.78 (0.88) | 0.04 |  |
|  | Mild/Moderate/Severe | 19 | 1.47 (1.50) |  | 14 | 1.79 (1.58) |  |  |
| 12. During the past 7 days, how bad was your worst bruising? | Normal | 13 | 1.08 (1.32) | 0.42 | 18 | 0.78 (1.48) | 0.04 |  |
|  | Mild/Moderate/Severe | 19 | 1.63 (2.45) |  | 14 | 2.71 (2.95) |  |  |
| 13. During the past 7 days, how often did you feel depressed? | Normal | 13 | 0.38 (0.87) | 0.02 | 18 | 0.61 (0.70) | 0.05 |  |
|  | Mild/Moderate/Severe | 18 | 1.39 (1.29) |  | 14 | 1.57 (1.55) |  |  |
| 14. During the past 7 days, how often did you feel angry? | Normal | 13 | 0.69 (0.75) | 0.12 | 18 | 0.50 (0.62) | 0.01 |  |
|  | Mild/Moderate/Severe | 19 | 1.16 (0.90) |  | 13 | 1.62 (1.33) |  |  |
| 15. During the past 7 days, how often did you feel irritable? | Normal | 13 | 1.15 (0.90) | 0.16 | 18 | 0.89 (0.58) | 0.00 |  |
|  | Mild/Moderate/Severe | 19 | 1.63 (0.96) |  | 13 | 2.15 (1.14) |  |  |
| 16. During the past 7 days, how often did you feel frustrated? | Normal | 13 | 0.92 (0.76) | 0.12 | 18 | 0.89 (0.68) | 0.01 |  |
|  | Mild/Moderate/Severe | 19 | 1.53 (1.35) |  | 14 | 2.21 (1.48) |  |  |
| 17. During the past 7 days, how often did you feel anxious? | Normal | 13 | 0.77 (1.09) | 0.00 | 18 | 0.78 (1.06) | 0.06 |  |
|  | Mild/Moderate/Severe | 19 | 2.11 (1.37) |  | 14 | 1.57 (1.22) |  |  |
| 18. During the past 7 days, how often did you experience mood swings? | Normal | 13 | 0.54 (0.52) | 0.02 | 18 | 0.44 (0.62) | 0.02 |  |
|  | Mild/Moderate/Severe | 19 | 1.37 (1.26) |  | 14 | 1.64 (1.60) |  |  |
| 19. During the past 7 days, how much were your daily activities limited? | Normal | 13 | 0.46 (0.66) | 0.07 | 18 | 0.17 (0.38) | 0.00 |  |
|  | Mild/Moderate/Severe | 19 | 1.06 (1.09) |  | 14 | 1.50 (1.40) |  |  |
| 21. During the past 2 weeks, how much did you worry there might be problems resulting from your treatment for TTP? | Normal | 13 | 0.00 (0.00) | 0.01 | 13 | 0.15 (0.38) | 0.01 |  |
|  | Mild/Moderate/Severe | 19 | 1.20 (1.42) |  | 10 | 1.50 (1.27) |  |  |
| 22. During the past 2 weeks, how bad was your allergic reaction to your treatment for TTP? | Normal | 13 | 0.00 (0.00) | 0.02 | 13 | 0.00 (0.00) | 0.10 |  |
|  | Mild/Moderate/Severe | 19 | 1.31 (1.92) |  | 10 | 1.00 (1.70) |  |  |
| 23. During the past 2 weeks, how bad was your drowsiness related to your treatment for TTP? | Normal | 13 | 1.13 (2.47) | 0.01 | 13 | 1.15 (2.12) | 0.00 |  |
|  | Mild/Moderate/Severe | 19 | 4.50 (3.10) |  | 10 | 4.80 (2.70) |  |  |
| 24. During the past 2 weeks, how bad was your pain due to injection, IV insertion, or port access for your TTP treatment? | Normal | 13 | 1.13 (1.64) | 0.29 | 13 | 1.08 (1.32) | 0.10 |  |
|  | Mild/Moderate/Severe | 19 | 2.00 (2.25) |  | 10 | 3.20 (3.58) |  |  |
| 26. During the past 2 weeks, how much did the travel to receive your treatment for TTP feel like a burden (i.e., the actual distance or getting someone to go with you)? | Normal | 13 | 1.38 (1.06) | 0.82 | 13 | 1.08 (0.86) | 0.97 |  |
|  | Mild/Moderate/Severe | 19 | 1.25 (1.48) |  | 10 | 1.10 (1.45) |  |  |
| **Domain scores/Total score** | | | | | | | |  |
| cTTP Total Fatigue Score | Normal | 13 | 3.08 (2.96)  5.37 (2.87) | 0.04 | 17 | 2.65 (1.93) | 0.04 |  |
|  | Mild/Moderate/Severe | 19 |  |  | 14 | 4.64 (3.15) |  |  |
| cTTP Total Limited Activities Score | Normal | 13 | 0.46 (0.66) | 0.07 | 18 | 0.17 (0.38) | 0.00 |  |
|  | Mild/Moderate/Severe | 17 | 1.06 (1.09) |  | 14 | 1.50 (1.40) |  |  |
| cTTP Total Headache Score | Normal | 13 | 0.85 (0.90) | 0.15 | 18 | 0.78 (0.88) | 0.03 |  |
|  | Mild/Moderate/Severe | 19 | 1.47 (1.50) |  | 14 | 1.79 (1.58) |  |  |
| cTTP Pain/Bruising Domain Score | Normal | 13 | 3.92 (3.97) | 0.01 | 18 | 3.39 (3.13) | 0.00 |  |
|  | Mild/Moderate/Severe | 19 | 10.42 (9.61) |  | 14 | 13.36 (9.44) |  |  |
| cTTP Cognitive Impairment Domain Score | Normal | 13 | 2.23 (1.42) | 0.01 | 18 | 2.06 (1.92) | 0.01 |  |
|  | Mild/Moderate/Severe | 19 | 4.53 (3.13) |  | 14 | 4.79 (3.58) |  |  |
| cTTP Visual Impairment Domain Score | Normal  Mild/Moderate/Severe | 13  19 | 0.54 (0.88) | 0.01 | 18  14 | 0.33 (0.69)  2.93 (3.02) | 0.00 |  |
|  |  |  | 2.47 (2.99) |  |  |  |  |  |
| cTTP Mood Domain Score | Normal | 13 | 4.46 (2.67) | 0.01 | 18 | 4.11 (2.97) | 0.00 |  |
|  | Mild/Moderate/Severe | 19 | 9.24 (5.5) |  | 14 | 10.71 (6.84) |  |  |
| cTTP Treatment Burden Score | Normal | 8 | 3.63 (4.17) | 0.01 | 13 | 3.46 (2.26) | 0.00 |  |
|  | Mild/Moderate/Severe | 16 | 10.38 (7.62) |  | 10 | 11.60 (7.07) |  |  |
| cTTP Total Score | Normal | 13 | 18.71 (8.24) | 0.00 | 18 | 16.39 (7.49) | <0.0001 |  |
|  | Mild/Moderate/Severe | 19 | 44.99 (26.98) |  | 14 | 49.66 (23.81) |  |  |
|  |  |  |  |  |  |  |  |  |

PGIS response categories 'mild', 'moderate', and 'severe' were combined to form one category and compared with response category 'normal.' Items 20 and 25 were excluded from total scoring and psychometric analyses and so are not presented in the above table.

This analysis is exploratory given the lack of variability found in the descriptive analysis results.

The original PGIS categories could be used as an option instead creating two groups, however 80% of participants endorsed normal or mild in severity.

*cTTP* Congenital thrombotic thrombocytopenic purpura, *PGIS* Patient Global Impression of Severity, *SD* Standard deviation, *TTP* Thrombotic thrombocytopenic purpura
